# The Designing, Testing, and Utility of a 3D-Printed Respirator: A Hospital's Journey Into Self-Sustainability During COVID-19

**DOI:** 10.7759/cureus.18113

**Published:** 2021-09-20

**Authors:** Aditya Lal Vallath, Ravisha More, Satyajeet Bhaskare, Sarabjeet Rattan, Ajinkya Athlye, Adheeth Praveen, Bindi S Patel, Vyom Richharia, Akshita Lalendran, Sudhir Patsute

**Affiliations:** 1 Emergency Medicine, Peerless Hospital and B.K. Roy Research Center, Kolkata, IND; 2 Infectious Disease, Dr. Naidu Infectious Diseases Hospital, Pune, IND; 3 3D Printing, SkaizenTech LLC, Pune, IND; 4 Statistics, GS Labs, Pune, IND; 5 Statistics, Independent, Kochi, IND; 6 Obstetrics and Gynaecology, Spectrum Health Butterworth Hospital, Michigan, USA; 7 Psychiatry and Behavioral Sciences, BronxMedcare Hospital, New York, USA; 8 Public Health, Dr. Naidu Infectious Diseases Hospital, Pune, IND

**Keywords:** personal protective equipment (ppe), infection control measures, ‎3d printing, covid 19, operations and supply chain management, research in emergency medicine, n95 respirators

## Abstract

Objective

The current global COVID-19 pandemic has disrupted supply chains and the production of essential goods and services. This includes personal protective equipment (PPE) kits, respirators, and other protective devices. Hence efforts were made to prototype and produce 3D-printed N95 respirators to fill the gap in supply. In addition, methods of sterilization were put into place for the respirators. As well as forming standard operating procedures.

Methods

With the use of vast open-source libraries and collaboration with engineers and doctors fighting the COVID-19 pandemic, respirator prototypes were produced with special consideration to the sizing to fit median facial sizes. Polymer plastics were mixed in various proportions to condition the respirator to be used by frontline workers in austere environments. Due to the shortage of medical-grade filter media, alternative sources were researched. Merv 13 and Merv 15 filters were selected due to their cheap costs, vast abundance, and proven filtration efficacy against particles of 0.03 microns. Studies conducted around the world have also shown its efficacy as an alternative to medical-grade air filter media. After developing standard operating procedures (SOPs) for sterilisation and respirator usage. Emergency approval was obtained and a limited number of healthcare workers were issued with this respirator (n=400). PPE kit satisfaction and self-efficacy scores were calculated from daily questionnaires during donning and doffing

Results

Qualitative fit-tests in all 400 healthcare workers matched those of a conventional N95 respirator. Almost all of the respondents in the PPE kit satisfaction responded positively. The self-efficacy score calculated from the general self-efficiency scale had an overall positive value, with the average score being 4.29. This demonstrated that the self-efficacy score was above average and indicated a high motivation to overcome obstacles and spend more time solving problems. The average self-efficacy score is defined between 2.5 - 3.5, and a low self-efficacy score is defined as a score below 2.5. Lastly, a regression analysis was done to test the correlation between PPE kit satisfaction and self-efficiency this demonstrated a positive correlation between PPE kit satisfaction using the 3D-printed respirator and self-efficacy (Slope: 0.416, Intercept: -1.066, R-value: 0.872, P-value: <0.01)

Conclusions

With supply chain disruptions and reduced or nonexistent supplies of essential medical goods. The need of a reusable, sterilisable, and efficient respirator has never been more evident. The materials used have made it sustain heavy use in austere environments. Studies have reported higher than average burnout rates in COVID-19-based healthcare workers. Studies have also shown that the rates of burnout are high in healthcare professionals without access to proper PPE kits in developing nations. This respirator was rated highly in PPE kit satisfaction and the self-efficacy score. Studies have demonstrated a correlation between high self-efficacy scores and low burnout rates in health care workers. There is also documented evidence of a positive correlation between high self-efficacy scores and general health. As the pandemic continues to evolve, so will the efforts to combat it, such as 3D printing. Interdisciplinary collaboration continues to drive our efforts to combat the pandemic and hopefully resolve it in the future.

## Introduction

The 2019 novel coronavirus disease (COVID-19) is a highly infectious viral disease that can cause a broad spectrum of symptoms in individuals infected with the virus, ranging from asymptomatic infections to severe acute respiratory illness. The illness may later progress to acute respiratory distress syndrome, sepsis, multi-organ failure, and eventually death [1,2]. The virus is highly infectious and is mainly spread via respiratory droplets and contact with contaminated surfaces [3]. During the disease, studies conducted worldwide demonstrate patients developing acute respiratory distress syndrome (ARDS) and other severe lung pathologies. These patients require respiratory support devices to prevent further deterioration and mortality. These respiratory support devices, such as ventilators and bilevel positive airway pressure (BiPAP) devices, generate substantial amounts of aerosol, which is detrimental to both patients and healthcare staff [4].

As the global disruption of supply chains worsened efforts by healthcare workers due to logistical challenges owing to disruptions in manufacturing and transportation, in this context, 3D printing has redeployed its capabilities in the crucible of COVID-19 responses, demonstrating its competitive advantage in this pandemic. The versatility and rapid prototyping of 3D printing empower a swift mobilisation of the technology and a rapid response to emergencies. Even during severe disruptions in supply chains, critical parts can be manufactured on-demand by any decentralized 3D-printing facility in the world by leveraging designs shared online. Moreover, the additive nature of 3D printing enables product customization and complex structures. The broad spectrum of 3D-printing applications in the fight against COVID-19 includes personal protective equipment (PPE), medical and testing devices, personal accessories, visualization aids, and emergency dwellings. 3D printers work on a method known as the layered production or additive manufacturing (AM) method. It utilizes complex 3D geometries using a computer-aided design (CAD) model. The printing process includes several steps such as prototyping, software flow, material analysis, printing, finishing, process validation, and testing. First, the desired printing model is designed using a CAD model. This CAD model is then converted into a file containing instructions on how the printer will create the product and sent it to the printer. Finally, the instructions are ready to be delivered to the printer as a printable software file (most commonly G-code [Massachussetts Institute of Technology, Cambridge, USA] or AMF [additive manufacturing file format]). After printing the product, post-processing steps and some testing can be applied to the product [5].

On this basis, 3D printing of respirators was concluded as the need of the hour. A qualitative fit-test was done to test its efficacy. The respirators were distributed amongst the health care staff of multiple government hospitals in Pune, which are designated as high-volume COVID-19 tertiary-care hospitals. However, the pandemic has proven to contribute to high levels of stress and mental fatigue in health care workers mainly due to the improper PPE kits or lack of access to them. Hence operator satisfaction was also defined as an essential need when producing these respirators. A survey was conducted to gauge operator satisfaction with the respirator and ascertain self-efficacy score, a measure of personal motivation in a challenging work environment.

## Materials and methods

Overview

The project was approved under Emergency IRB conducted at Dr. Naidu Infectious Diseases Hospital (IRB number: PMC4140250220). With the availability of exhaustive 3D printing libraries on the internet, each 3D model was created to act as a sealed respirator that supported a filtration cartridge. Each design requires a filtration unit and a sealed full-face design. Most of the available plans were sourced online via open source communities such as Makerspace and Reddit. The files are available in .stl or .cad file formats. The files were edited using programs such as Meshmixer (Autodesk Inc, San Rafael, USA) to adapt to the needs and changes as they arrived dynamically. The models were chosen due to their popularity and use amongst clinicians around the world [6-8]. In addition, they were considered after recognizing the challenges faced in low-resource, high-volume hospitals. The masks and accessories that were chosen are listed in Table 1.

**Table 1:** Different prototypes and their properties

Option	Name	Source	Consideration
Option 1	Wiles COVID Pandemic Mask	https://www.thingiverse.com/thing:4237783	The mask is simple to print and assemble due to its minimalistic design.
Option 2	The Montana Mask	https://www.makethemasks.com/	The mask is simple to print and assemble. Extensive clinical testing has been done to prove its efficacy.
Option 3	Nanohack 2.0	https://copper3d.com/hackthepandemic/	The mask has built-in exhaust valves, which make using the mask comfortable for extended periods.

After a careful review of the literature and results of fit-tests done worldwide, Option 2, Montana Mask, was selected for use in our setting due to its simplicity and extensive testing regarding the fit-test and efficacy. This respirator was built upon a distribution under an open-source license (Creative Commons Attribution 4.0 International License, CC BY 4.0). The sizes of the respirator printed were small, medium, and large. The dimensions were calculated using data sourced from a study conducted by National Institute for Occupational Safety and Health (NIOSH), which formed the median measurements by measuring anthropometric data from different populations [9-10]

3D filaments

A 3D filament is a primary material 3D printers use to form solid objects. There is a wide variety of materials used in 3D printing; however, we utilized the most commonly available source to keep production costs low. These materials are polylactic acid (PLA), polyethylene terephthalate glycol (PETG), and acrylonitrile butadiene styrene (ABS). The properties of the materials are listed in Table 2.

**Table 2:** Physical properties of the materials PLA: polyactic acid; PETG: polyethylene terephthalate glycol; ABS: acrylonitrile butadiene styrene

S.no	Name	Heat resistance	Tensile strength	Tensile Modulus	Heat Deflection Temperature
1.	PLA	X	37 MPa	2.7–16 GPa	49-52°C
2.	ABS	X	40 MPa	1.4 - 3.1 Gpa	97°C
3.	PETG	X	53MPa	2.2Gpa	64°C

A decision was taken after observing drawbacks in the respirator construction when a single type of plastic was used. A combination of the different plastic filaments was used best to utilize each of the plastics' full potential. PETG was used in the construction of the respirator's main body due to tensile strength. PLA was used in the filter locks due to its rigidity and high tensile modulus to combat high shear stress forces during locking of the filters, and ABS was used to make the head harness due to its flexibility. All filaments are manufactured by Fillamentum and produced in the Czech Republic (Fillamentum Manufacturing Czech s.r.o., Hulin, Czech Republic).

Equipment - 3D printers

The respirators were printed using Prusa i3 MK3S (Prusa Research, Prague, Czech Republic), and the method of printing was Fused deposition modeling (FDM) printing. The FDM method uses printing layers of product thermoplastic polymer filament. The filament is heated until a semi-liquid state is reached at the nozzle and then extruded onto previously printed layers. This method is cost-effective and easy to use. It can be printed by FDM printers using various materials such as PLA, ABS, and thermoplastic elastomer/thermoplastic polyurethane (TPE/TPU). The extruder size was 0.4mm, and the bed dimensions were length 210mm x width 250mm x height 340mm.

Filtration capabilities

The N95 respirators are designed to filter 95% of particles 0.3 microns in size to protect the user from infectious aerosols. However, there has been a shortage due to the ongoing pandemic because of manufacturing and supply chains disruption. As a result, many studies have shown that minimum efficiency reporting value (MERV) filters are ideal and equal in efficiency to the N95 respirator. In this study, we have used the combination of a double-pleated MERV 13 filter and MERV 15 filter [11] manufactured by LydAir (Lydall Performance Materials, Manchester, USA) and supplied by United Filters, Pune, India. MERV is expressed on a 20 point scale and is derived from filtration's particle size efficiency (PSE). Examples of MERV designations, air filter uses, and filtration efficiencies are listed in Table 3. 

**Table 3:** MERV rating MERV: minimum efficiency reporting value; blds: buildings

Standard 52.5 Minimum Efficiency Reporting Value	Dust spot Efficiency	Arrestance	Typical Controlled Contaminant	Typical Applications	Typical Air Filter/Cleaner Type
20 19 18 17	N/A	N/A	<0.30 pm particle size virus(unattached) carbon Dust All combustion smoke	Cleanrooms RadioactiveMaterials PharmaceuticalMan. CarcinogenicMaterials	>99.99%- .10-.20 pm particles >99.99%- 30 pm particles
16 15 14 13	N/A >95% 90-95% 89-90%	N/A N/A >98% >98%	.30-1.0pm particlesize All Bacteria Most Tobacco smoke proplet Nuclei	General Surgery Hospital Inpatient care smoking Lounges commercial blds	Bag Filter non supported Box filter
12 11 10 9	70-75% 60-65% 50-55% 40-45%	>95% >95% >95% >90%	1.0-10.0 pm Particle Size Legionella Humidifer Dust lead Dust milled flour, Auto emission	residential blds commercial blds	Bag filters Box Filters
8 7 6 5	30-35% 25-30% <20% <20%	>90% 90% 85-90% 80-85%	3.0-10 pm particle size Mold spores fabric protector Cement dust	Industrial workplace Paint booth	Pleated Filters Cartridge filters Throwaway filters
4 3 2 1	<20% <20% <20% <20%	75-80% 70-75% 65-70% <65%	>10.0 pm Particle size pollen Sanding Dust textile fibers	Minimal filtration Window A/c Units	Throwaway Filters Washable filters Electrostatic filters

Based on the above specifications, it is conceivable that MERV 15 filters may be a candidate replacement for the N95 filter, and MERV 11-16 material filters may also be viable if enough layers of material are used. For our filters, a layer of MERV 15 was sandwiched between two layers of MERV13 to increase filtration capacity. As a result, the combination of MERV filters offers filtration of a typically controlled contaminant of 0.30 - 1.0 pm in size and provides sufficient filtration for COVID-19 virus particles, each 50-200 nanometres in diameter [12].

Fit-testing protocol

The fit-test protocol was conducted to test the efficiency of the respirator and compare its efficacy as a viable reusable candidate for a conventional N95 respirator. All potential users of the mask had to be screened to ensure they could fulfill the testing protocol. The qualitative fit-test protocol was conducted using a freshly prepared saccharin solution, which can be used as an alternative to commercially available fit-tests that were not freely available due to pandemic supply chain disruptions. Homemade saccharin for fit-tests has shown to be close in its sensitivity and specificity to commercial solutions; hence it was prepared by dissolving 830mg of sodium saccharin in 100mL of warm water [13]. The steps for the qualitative fit-test were adapted from NIOSH.

Sterilisation methods

Due to the highly infectious nature of COVID-19 and its ability to remain viable on surfaces such as plastics for up to 80 hours [14], it was of the utmost importance to have safe and effective sterilization methods. One of the problems encountered was the porosity of conventional PLA plastic, one of the critical materials used in 3D printing the respirator. This was rectified by adding ABS plastic in ratio with PLA plastic, thereby reducing the porosity of the respirator. Disinfection methods for the body and filter holder of the mask were designed with reference to the WHO biosafety handbook. 0.54% sodium hypochlorite (NaOCl) solution was decided to be the optimal disinfection solution due to its availability and affordability [14][15]. The biocidal effects are well documented, and disinfection methods were designed from it. The operator removed the filters from the respirator after donning the necessary PPE Kits. The respirator body and the filter holder were wiped clean with a clean disposable paper towel soaked in freshly prepared 0.54% NaOCL solution for 15 seconds; it was allowed to air dry and then stored in dry labeled paper bags. The frequency of disinfection was three days.

Due to these graded MERV filters in heavy industry, these filters were sterilized by Ultraviolet Germicidal Irradiation (UVGI) radiation. This method was proven to be effective as a viricide and safe as it does not damage the structural integrity of the filter. A dose of 300 mJ/cm2 for 15 mins at 1.2 meters away from the UVGI source was found to be effective [16-18]. This method of sterilization was used for the filters after three days of use. Between the three days of use, the respirator is placed in a clean, dry paper bag with the filter side facing up when not used and stored in a dry room. After UVGI sterilization, the filters were packed into clean and labeled dry paper bags and re-issued to their original user. The filters were discarded if -

1. There any structural deformities in the fibers of the filter 2. The user reports difficulty breathing through the filters 3. If the filters are heavily soiled with debris 4. Three cycles of UVGI sterilization have already been used on the filter

Usage and self-efficacy

After setting up protocols for safe use and methods of sterilization were introduced. The respirators were introduced to limited staff (N=400) after Emergency Ethical Approval was confirmed. The respirators were distributed to health care workers who could satisfy the requirements of the fit-test. This included nurses, doctors, laboratory technicians, support staff, social workers, and auxiliary staff. They were instructed on the correct usage and methods for sterilization and disinfection of the mask and were issued the appropriately sized respirators for their use. Daily charting of usage and PPE satisfaction was done with the help of logbooks during the donning and doffing procedures. PPE satisfaction was gauged with a simple Yes/No questionnaire [19] to the questions mentioned in Table 4; "Yes" was given a score of 1, and "No" was given a score of 0. The average score was calculated by taking all the questions divided by the total number of questions.

**Table 4:** PPE Kit satisfaction survey PPE: personal protective equipment

1.	Do you wear PPE during duty?	Yes=1/No=0
2.	Are you satisfied with the respirator provided to you?	Yes=1/No=0
3.	Are you able to do your duties satisfactorily with the respirator?	Yes=1/No=0
4.	Are you satisfied with the sterilization methods used on the respirator and its filter?	Yes=1/No=0
5.	Are you confident in using this respirator during your duties?	Yes=1/No=0
6.	If your answer is no in any of the following questions, please mention the reason for it below to rectify it.	

Self-efficacy was defined as the ability of oneself to execute courses of action required to deal with prospective and demanding situations [20]. As a result, it has become a valuable indicator in measuring job-related satisfaction and work-related performance. Studies have shown that overall positive self-efficacy has been related to better job performance. In addition, individuals with higher self-efficacy make more effort to complete tasks in challenging/austere environments than individuals with lower self-efficacy [21]. Self-efficacy was measured with the new general self-efficiency scale developed by Stanford University and SPARQtools (www.sparqtools.org/). It is an eight-item measure with a 5-point rating scale, shown in Table 5. Each individual's results were averaged, calculated by adding respondents' answers to each item and dividing this sum by the total number of items [21]. The new general self-efficacy scale is listed in Table 5.

**Table 5:** The new general self-efficiency scale

1.	I will be able to achieve most of the goals that I set for myself.	1 = strongly disagree	2 = disagree	3 = neither agree nor disagree	4 = agree	5 = strongly agree
2.	When facing difficult tasks, I am certain that I will accomplish them	1 = strongly disagree	2 = disagree	3 = neither agree nor disagree	4 = agree	5 = strongly agree

3.	In general, I think that I can obtain outcomes that are important to me	1 = strongly disagree	2 = disagree	3 = neither agree nor disagree	4 = agree	5 = strongly agree
4.	I believe I can succeed at most any endeavor to which I set my mind	1 = strongly disagree	2 = disagree	3 = neither agree nor disagree	4 = agree	5 = strongly agree
5.	I will be able to successfully overcome many challenges	1 = strongly disagree	2 = disagree	3 = neither agree nor disagree	4 = agree	5 = strongly agree
6.	I am confident that I can perform effectively on many different tasks	1 = strongly disagree	2 = disagree	3 = neither agree nor disagree	4 = agree	5 = strongly agree
7.	Compared to other people, I can do most tasks very well	1 = strongly disagree	2 = disagree	3 = neither agree nor disagree	4 = agree	5 = strongly agree
8.	Even when things are tough, I can perform quite well	1 = strongly disagree	2 = disagree	3 = neither agree nor disagree	4 = agree	5 = strongly agree

Finally, a regression analysis of the data was conducted to test the correlation between PPE kit satisfaction and self-efficacy. This was done to test the hypothesis that PPE kit satisfaction is related to higher levels of self-efficacy, which is essential for the high-stress environment of a dedicated COVID-19 tertiary care hospital.

Statistical analysis

Statistical analysis was done on the Ubuntu 18 operating system (www.ubuntu.com) using open-source python libraries. Regression analysis was done on Scikit-learn 0.23.1 (www.scikit-learn.org/) [22] with plotting calculation and represented with Matplotlib 3.2.2 (www.matplotlib.org/) [23], JupyterLabs 2.2.2 (www.jupyter.org/), pandas 1.0.5 (https://pandas.pydata.org/) [24]. 

## Results

**Figure 1:**
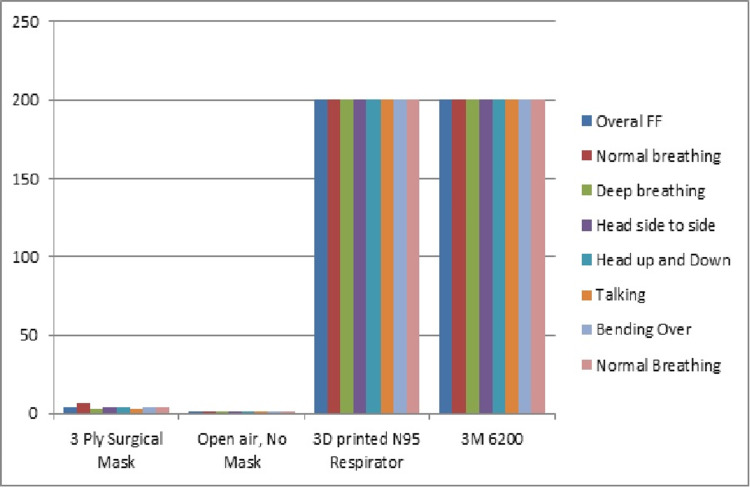
Fit-test results The graph represents pass rates for the fit-test excercise. X-axis: type of mask; Y-axis: fit test score

**Figure 2:**
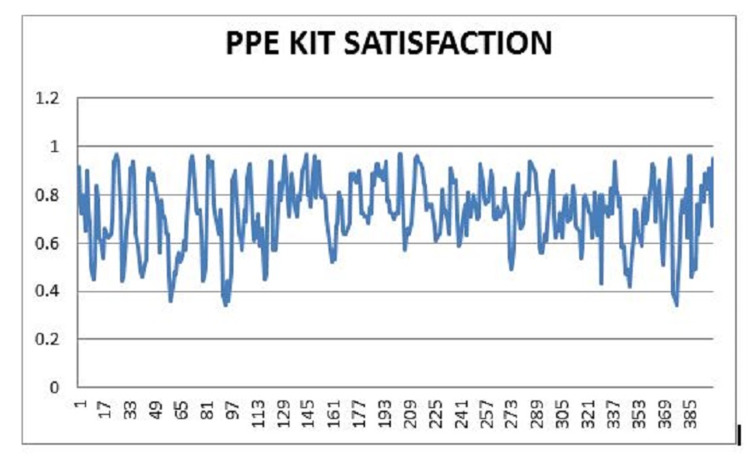
PPE kit satisfaction X-axis: PPE kit satisfaction; Y-axis: questionnaire participants PPE: personal protective equipment

Health care workers did the qualitative fit-test before being issued the respirator. The fit-test was done for the respirator and was compared with conventional surgical masks and other respirator designs. The results were satisfactory and matched the results published by the original creators of the respirator [25], as seen in Figure 1. Provisions were made in the event of any negative deviation in the fit-test, with either issuing an appropriately sized respirator or asking the user to shave off facial hair or remove jewelry from the face to maintain the seal. Filters were also replaced if there was a deviation in the fit-test. PPE kit usage and satisfaction were measured with the help of questionnaires during the donning and doffing procedures. The results are detailed in Figure 2.

**Figure 3:**
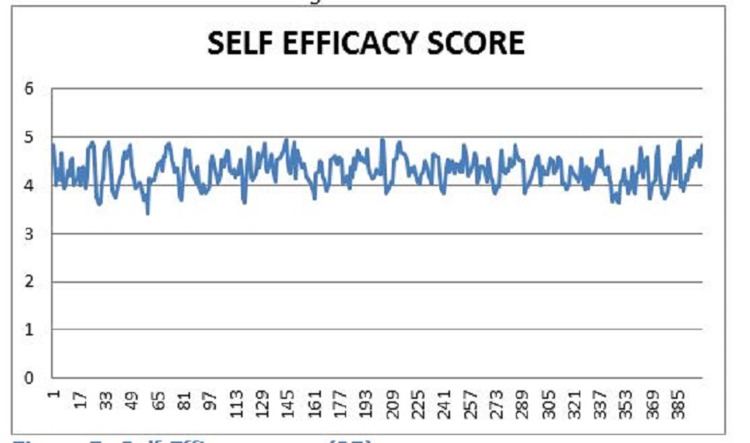
Self-efficacy score X-axis: self-efficacy score; Y-axis: questionnaire participants

Although the majority of the respondents answered positively in the questionnaire, some had concerns facing the respirator. One of the main concerns of the respirator was the muffling and dampening of the user's voice when the respirator was in use. Users have stated that they need to increase their voice and volume to overcome this problem, leading to voice strain. Another issue brought up by the users is the built-up of humidity and heat in the mask, which led to discomfort in users over extended periods; plans to develop a solution to mitigate this problem are currently underway. Self-efficacy was calculated with the new general self-efficiency scale [21]. The results are shown in Figure 3.

**Figure 4:**
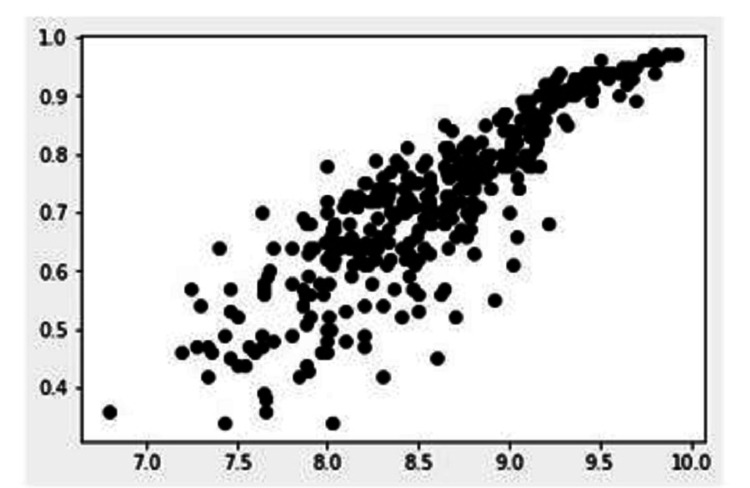
Linear Regression model between PPE Kit satisfaction and Self Efficacy X-axis: self-efficacy score; Y-axis: PPE kit satisfaction PPE: personal protective equipment

**Figure 5:**
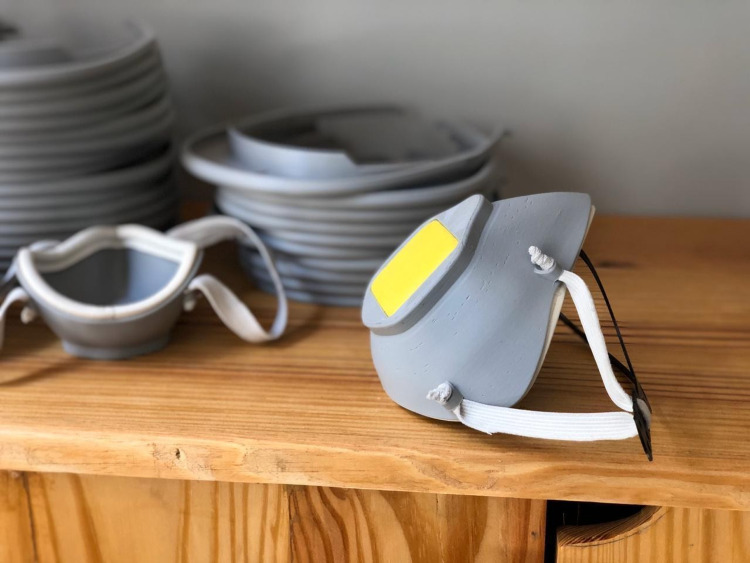
3D-printed respirator with filter piece

**Figure 6:**
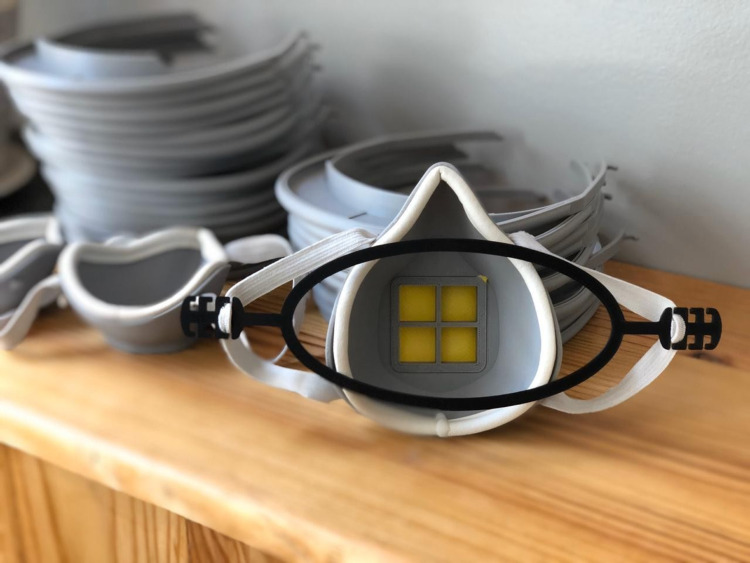
3D-printed respirator with filter piece

The self-efficacy score calculated from the general self-efficiency scale had an overall positive value, with the average score being 4.29. This demonstrated that the self-efficacy score was above average and indicated a high motivation to overcome obstacles and spend more time solving problems. The average self-efficacy score is defined between 2.5 - 3.5, and a low self-efficacy score is defined as a score below 2.5 [20]. Lastly, a regression analysis was done to test the correlation between PPE kit satisfaction and self-efficiency. The results are shown in Figure 4 and Table 6. The finished respirator is demonstrated in Figures 5, 6.

**Table 6:** Linear regression results

1.	Slope	0.416
2.	Intercept	-1.066
3.	R-value	0.872
4.	P-value	<0.01
5.	Standard Error	0.011

## Discussion

The COVID-19 pandemic has already put a massive strain on the manufacturing and supply chain for PPE and other equipment resulting in a shortage. Due to the decentralized nature of 3D printing and the vast sources of information available on the internet. Efforts can be made to cheaply produce highly customized solutions to the problems faced in the pandemic. These technologies are also improved with every iteration due to their open-source nature and worldwide collaboration.

It has been well documented that 3D printing technology has been used to mitigate challenges worldwide, such as rapidly and cheaply producing respirators, ventilator adapters, face shields, mask adapters, nasopharyngeal swabs, emergency quarantine facilities, training models, etc. A reusable respirator can mitigate some of the strain caused by the shortage of N95 respirators, especially in low-volume areas such as outpatient departments and wards. These masks can be safely sterilized using 70% ethanol solution, as previously used to disinfect surfaces from COVID-19. The filter pieces can be safely sterilized using UVB radiation, autoclaves, or conventional microwaves [26,27], all three of which are readily available in low-resource hospitals. These 3D-printed respirators can be produced and disseminated to healthcare settings where a robust supply chain is absent, protecting health care workers and conserving N95 respirators for higher risk settings, such as in aerosol-generating procedures in the ICU.

Although the merits of 3D printing are evident, standard regulations which govern most medical devices are loosely followed in 3D-printed devices. This is mainly due to these devices' decentralized nature and open-source nature; however, recently, U.S. government entities such as the U.S. Food and Drug Administration (FDA) and National Institutes of Health (NIH) have come up with a risk classification for 3D printed devices to meet safety and efficiency goals. All manufacturing and post-processing activities must be documented and performed within an ISO 13485-compliant quality management system. Final printed devices must be evaluated for biocompatibility based on international standards (such as ISO10993-1).

Building a solid framework for accountability and implementing guidelines supported by regulators and even governments can help establish greater confidence in 3D-printed medical devices [28]. The pandemic has also highlighted the mental health challenges faced by healthcare workers daily. Studies have reported higher than average burnout rates in COVID-19-based healthcare workers [29]. Studies have also shown that the rates of burnout are high in healthcare professionals without access to proper PPE kits in developing nations. [30] This respirator was rated highly in PPE kit satisfaction and the self-efficacy score. Studies have demonstrated a correlation between high self-efficacy scores and low burnout rates in health care workers [31]. There is also documented evidence of a positive correlation between high self-efficacy scores and general health.

## Conclusions

As the pandemic continues to evolve, So will the efforts to combat it, such as 3D printing. Interdisciplinary collaboration continues to drive efforts to combat the pandemic. COVID-19 pandemic will require decentralized and novel approaches to encounter challenges as they form. Further research is necessary to standardise and develop global standards to make production safer, quicker, and efficient for future pandemics.
